# Uptake of Voluntary Occupational Health Care—Assessments of German Occupational Health Physicians and Employees

**DOI:** 10.3390/ijerph19159602

**Published:** 2022-08-04

**Authors:** Martina Michaelis, Christine Preiser, Susanne Voelter-Mahlknecht, Nicole Blomberg, Monika A. Rieger

**Affiliations:** 1 Institute of Occupational and Social Medicine and Health Services Research, University Hospital, 72074 Tübingen, Germany; 2 Research Centre for Occupational and Social Medicine (FFAS), 79098 Freiburg, Germany; 3 Institute of Occupational Medicine, Charité—University Medicine, Corporate Member of Free University and Humboldt University, 13347 Berlin, Germany; 4 Statutory Accident Insurance and Prevention in the Health and Welfare Services (BGW), 44789 Bochum, Germany

**Keywords:** occupational health care, workplace health management, uptake of occupational health care, predictors for uptake, workers’ health surveillance, survey, interviews, multi-perspective study, occupational health physicians, employees

## Abstract

Workers’ health surveillance is considered essential for employees’ health and protection against hazardous working conditions. It is one part of occupational health care and thus one of four pillars of holistic workplace health management. In Germany, employers are obliged to provide mandatory and voluntary occupational health care (OHC) to employees, dependent on the defined occupational hazards. However, employees are not obliged to make use of voluntary OHC. No empirical information is available about the uptake of voluntary OHC by employees and the influencing factors in Germany. Thus, we carried out an explorative multi-perspective study with qualitative and quantitative elements to get insights from the view of occupational health physicians (OHPs) and employees. We conducted a survey among OHPs based on prior statements from two focus group discussions. A multivariate logistic regression analysis was performed to detect enablers and barriers regarding employee uptake of the offered voluntary OHC. We used extended qualitative methods among employees instead of an analogous survey. In total, 460 OHPs participated in the survey (response rate 29.1%), and 25 employees took part in interviews. Most of the employees had not heard the term voluntary OHC before, and only a few remembered respective occupational health care after explanatory request. In total, 78% of the OHPs assessed that employees always/mostly take up voluntary OHC. The most important attributed reason for non-uptake was that employees see no need for occupational health care when they feel healthy. The most important enabler for the perceived high uptake of voluntary OHC in the regression analysis was a positive attitude of the OHP toward voluntary OHC. While OHPs perceived that voluntary OHC was accepted by a majority of employees, this was not confirmed by the interviews with selected employees. This could indicate that the OHP respondents overestimated the amount of uptake. Since it became clear that employees are often unfamiliar with the terminology itself, we see a need for more and better information regarding the objectives and content of occupational health care to improve this important pillar of workplace health management.

## 1. Introduction

Workplace health management as a holistic strategy for the management of employees’ health is based on four pillars, usually referred to as “occupational safety and health”, “return-to work”, “workplace health promotion”, and “personnel development” [1]. Here, we focus on “occupational health care” (OHC), which stands for periodical appointments of employees with the occupational health physician (OHP) who gives advice on measures to prevent work-related and occupational diseases or a deterioration of work ability. During OHC, worker health surveillance (WHS) is offered to detect adverse health effects resulting from occupational exposures [2]. Occupational health care is a part of the first pillar—“occupational health and safety”.

The work of OHPs is considered essential for the prevention of occupational and work-related diseases [3,4], and the surveillance of employees’ health is implemented in European regulations [5] and international recommendations [6,7]. In Germany, as in other countries of the European Union, medical examinations and counselling by OHPs in the case of the hazardous risk potential of occupational activities are anchored in law [8,9,10].

In Germany, depending on the type of hazardous risk, OHC is either mandatory or voluntary for employees. Mandatory occupational health care must be provided in the case of, for example, being exposed to certain hazardous substances (e.g., cancerogenic substances) or certain biological agents categorized in higher risk groups. Voluntary occupational health care covers, for example, activities involving hazardous substances, if exposure cannot be ruled out, being exposed to biological agents categorized in lower risk groups, but also regularly working in wet conditions for more than two but less than four hours per day or working at visual display units (VDU) or a computer screen. In both cases, the employer is obliged to offer OHC to employees periodically.

According to the working conditions and general high standards of occupational safety and health in Germany, the number of employees entitled to receive voluntary OHC exceeds the number of those entitled to mandatory OHC (own estimation based on Barth et al. [11]). In general, the content of OHC addresses specific work-related hazards. General or special health check-ups at the workplace, on the other hand, are designed to prevent individual lifestyle-related diseases [12] and can be offered by the employer to all employees irrespective of specific occupational hazards [13] as a measure of workplace health promotion [1].

Despite the legal obligation for all employers in Germany to offer their employees access to an occupational health physician, not all employees have this opportunity. The reasons for this are the much-discussed general shortage of OHPs and the lack of contracts between employers and OHPs, especially in smaller enterprises [11,14].

The analysis of the uptake of health care and preventive health care measures has long been an important pillar of health care research in population-based studies in the secondary and tertiary prevention sector [15,16,17,18,19] as well as in the operational setting analyzing the uptake of occupational health services [20,21] or workplace health promotion [22,23,24]. In this context of health care research, we do not know enough about the extent to which employees take up (voluntary) occupational health care in Germany when it is offered. Thus, we started a comprehensive modular study considering diverse perspectives and using qualitative and quantitative approaches for data assessment. A detailed research report in German can be found online [25].

In the present analysis, we focus on survey results from the perspective of occupational health physicians and complementary statements of employees interviewed in focus groups. The following research questions were operationalized:

1. To what extent do employees make use of voluntary occupational health care (voluntary OHC)?
2. If employees do not follow the invitation for voluntary OHC, for what reasons?
3. Which aspects predict the uptake of voluntary OHC from a statistical point of view?

## 2. Materials and Methods

### 2.1. Research Design

Questionnaire items for a survey among OHPs were developed based on respective statements in two focus group discussions (*n* = 5 and *n* = 3) performed earlier. We used an explorative mixed-methods approach with a sequential phase model of the research process to generate a benefit from the qualitative process for the quantitative research process [26]. The qualitative research part included two focus groups (*n* = 5 OHPs, *n* = 4 OHPs and health and safety experts) and 26 expert interviews with representatives of a broad range of institutions (works councils, employers’ associations, trade unions, statutory accident insurance institutions, state organization of labor protection), employers, and occupational safety specialists.

The item operationalization generated from the qualitative work was complemented by findings from a comprehensive systematic literature review focusing on German-language sources and performed by internet and manual search in subject-specific websites, journals, congress reports, paper-based information sources, discussion and question forums, and by means of the search engine Google in Scholar mode.

Aiming to focus on the experiences and attitudes of employees, we initially followed an analogous approach, i.e., focus group discussions followed by a survey. However, we encountered great difficulties in the course of planning the corresponding survey. An open internet survey was not possible because we would not have received reliable background information on the framework of occupational health care in the enterprise of each responding employee. Thus, we would have had to investigate employees of individual companies in a total survey and at the same time record the external framework conditions of occupational health care (OHC). This would have been rather demanding as in each company, the works council would have had to give its consent. This was in conflict with our intention of mapping a variety of industries as far as possible with the given time and funding of the research. Since a pronounced lack of familiarity with the OHC concept was already evident in the employee focus groups and interviews, we decided to extend data assessment using qualitative methods instead of a survey. By doing so, we were able to gain deeper insights into the experiences and attitudes of employees regarding occupational health care. Aiming to contrast these findings with the results of the data available from OHPs, in the following, we report the results of the OHPs’ survey and the employees’ interviews and focus group discussions sequentially.

### 2.2. Instruments

The questionnaire to investigate OHPs’ assessments was mainly self-developed. To answer research question 3 (predictors for the uptake of voluntary OHC), the established instrument FAGS on the general importance of occupational health and safety in the enterprise [27] was introduced as one of the factors possibly influencing the uptake of voluntary OHC (subscale “enterprise norms” with nine items and verbal transformation from “occupational safety” to “occupational health and safety”). For details of this scale and the following, see Appendix A Table A1.

The items describing (a) the role of OHPs as a possibly influencing factor and (b) the OHPs’ own attitudes toward voluntary OHC were based on brainstorming within the interdisciplinary team at the beginning of the project. The team consisted of sociologists experienced in work-related research questions, a safety engineer, and occupational health physicians. The items covered the legally defined tasks of OHPs and relevant aspects of the results, which were obtained from the qualitative data assessment carried out previously as described above (two focus group discussions with in total eight OHPs and one safety engineer).

Mean scores were constructed for all three dimensions. Enterprise-related descriptions were requested for the last enterprise the OHPs were in contact with.

Key questions in the interviews and focus groups of employees were, in summary, employers’ actions for employee health, experiences with OHPs, employees’ ideas regarding the term voluntary occupational health care, ideas for enablers and barriers for the employer to offer occupational health care and the uptake by employees, and finally suggestions for improvement (see Appendix A Table A2).

### 2.3. Samples and Recruitment

For multivariate analyses with the OHP data, at least 1000 persons needed to be contacted to attain a necessary sample size of at least *n* = 300 (plus surplus to compensate for missing values) and an expected response rate after one written reminder of 30% according to prior experiences [28]. After the questionnaire pretest, 1580 occupational health physicians were contacted for a paper survey in 2012. Specifically, they were approached with support of the Professional Association of German Occupational Health Physicians [29] in 8 out of 16 selected federal states of Germany, covering 11 out of 20 regions within the association.

To recruit employees, we applied convenience sampling strategies and distributed printed calls for participation at the local offices of a large statutory health insurance firm as a trustworthy institution in the field of health and in public spaces and published an advertisement in a local newspaper. Due to the sampling strategy, we had no further insights into the number of contacts and the number of drop-outs. A fee of 30 EUR was paid as an incentive for participation in an interview or focus group discussion.

### 2.4. Data Analysis

The OHP data evaluation was descriptive (percentages or mean, standard deviation (*SD*)) and excluded any missing values. Multivariate logistic regression analyses were used for research question 3 (“Which aspects predict the uptake of voluntary OHC?”). The effect size is expressed as the odds ratio (*OR*) with confidence interval (95% *CI*). To reach a parsimonious model, the backward method was used to eliminate variables from the model (*p_(out)_* = 0.6).

For this purpose, the outcome “uptake of voluntary OHC” was dichotomized (always/mostly vs. rarely/never). Missing values of the outcome (8.3%) and of the respective predictor variables were estimated by multiple imputations with IBM SPSS version 27 (five imputations, automatic method). The number of missing values of the predictor variables was generally below 5%, with the exception of “invitation to voluntary OHC in combination with other health care offers” (10.9%).

According to the exploratory approach, large numbers of variables were regarded as possible predictors for the outcome “uptake of voluntary OHC” and were analyzed exploratively. The variables covered several dimensions, namely, the framework conditions in the enterprise for occupational health and safety, the characteristics of occupational health care, information management about voluntary OHC, perceptions of OHPs, and structural characteristics of the enterprise. Details can be found in Appendix A Table A3.

The interviews and focus group discussions with employees took place between May and October 2012 and were carried out by CP. Saturation of the sample size was reached when no more fundamentally new topics were generated. The data were audio-recorded and then transcribed. All data were analyzed with qualitative content analysis [30]. The main categories were built deductively from the interview guide and research literature. The subcategories were built inductively from the data. To ensure quality control, all transcripts were analyzed by at least CP and a student assistant independently from each other and then discussed together. All other members of the interdisciplinary research team gave feedback to preliminary results at various stages of the process.

## 3. Results

### 3.1. Response Rates

The overall response rate in the *Occupational health physicians’ survey* was 29.1% (*n* = 460). Accepting a proportion of missing values in variables below 10%, the data quality of the results described in the following was good to sufficient. Only the items “uptake of voluntary OHC in case of simultaneous invitation to mandatory occupational health care” (missing values/answer “I don’t know”—16%) and items answering “reasons for missing uptake of voluntary OHC” (research question 2; partly > 10%) showed a higher rate of missing values.

In total, 24 employees working in small and medium-sized or large enterprises were included in the study. Two participants were interviewed individually; the other took part in five focus group discussions with 3, 4, 4, 5, and 6 persons, respectively.

### 3.2. Sample Characteristics (OHP Survey)

#### 3.2.1. Personal Characteristics

On average, the OHPs were 52 years old (*SD* ± 7) with work experience of 18 ± 8 years; 46% were women, 79% worked full-time, 52% supervised at least 11 enterprises, and 36% were permanently employed in the enterprise to which the information in the questionnaire related. A positive self-assessment regarding the role of the OHP in the respective enterprise was confirmed by 78–98% in 10 out of 12 items (score mean value 2.29 (*SD* 0.63) ranging from 0 to 3). The respondent’s attitude toward voluntary OHC was 1.70 (*SD* 0.31), with a score ranging from 0 to 3 (the higher the value, the better the attitude). More details can be found in Table 1.

#### 3.2.2. Characteristics of the Enterprise

When supervising more than one enterprise, respondents were asked to describe the situation only in the most recently supervised case. The enterprises to which the information in the questionnaire referred had been supervised by the respondents for an average of 10 years (*SD* 8).

In total, 47% of the 454 enterprises belonged to the production or processing industry; 78% had at least 250 employees. Usually, the enterprises had a quality management system, a works council, and good framework conditions for occupational health and safety (e.g., OHS committee, return-to-work management, risk assessment systems). Nearly all of them provided voluntary OHC (98%; for more details see Appendix A Table A4).

#### 3.2.3. Framework Conditions of the Occupational Health Care in the Enterprise

In 86% of the described enterprises, the OHP’s practice was more or less easily accessible for employees, being at most no more than two kilometers away from the workplace. In total, 55% of the practices could be consulted spontaneously. For more details, see Table 2.

#### 3.2.4. Characteristics of Information Management about Voluntary OHC

In summary, in 81% of the described enterprises, employees were generally informed about occupational health care during occupational safety training and in company operating instructions.

Nearly all OHPs (95%) reported that employees received a targeted invitation to a voluntary OHC by personal letter, e-mail or orally, mostly (88%) when invited in combination with other health care offers (mandatory OHC).

According the OHPs, on average, the invitation regarding voluntary OHC fulfilled 3.54 (*SD* 1.9) of the six legally required aspects. Such conditions include, for example, that the employee will not suffer any disadvantages if the offer is rejected or that the employer will not receive any information about the result of the appointment. Details can be found in Table 3.

In total, 32% of the OHPs reported that medical confidentiality was already addressed in the invitation and not only during the appointment, and 69% mentioned that the employer does not receive any individual results from the appointment.

### 3.3. Sample Characteristics (Employees)

Nine employees worked in the public sector, 12 in the private sector, two in the industrial sector, and one worked in craft business. In total, 13 participants were female. The age group distribution was 20–30 (*n* = 4), 30–40 (*n* = 5), 40–50 (*n* = 9), and 50–60 years (*n* = 6). The groups generated were heterogeneous within themselves in terms of age, sex, and professional activities and experiences. Details can be found in Appendix A Table A5.

### 3.4. Research Question 1: Uptake of Voluntary Occupational Health Care by Employees

#### 3.4.1. Occupational Health Physicians

A total of 78% of the occupational health physicians indicated that voluntary OHC was made use of “always” or “mostly” by the employees if it was provided by the employer of the enterprise to which the information in the questionnaire was related. Furthermore, nearly all OHPs assessed that employee uptake of voluntary OHC was higher when the offer was made in conjunction with a call to a mandatory OHC (see Table 4).

#### 3.4.2. Employees

In general, the level of information about voluntary OHC varied widely between the 25 employees. Few of them had profound knowledge about the various terms related to occupational health care.

Most employees had not heard of the terms before; moreover, many had never had personal contact to an OHP. Nevertheless, during the course of the discussion, some participants remembered the content of voluntary OHC appointments and reported that they had actually experienced voluntary OHC without specifically knowing what it was (exemplary quotations see Appendix A Table A6).

Some also reported that they were not able to distinguish between mandatory and voluntary parts at the time of the appointment, especially when both were integrated into the same appointment. Some employees spoke of specific days or time slots during which certain voluntary OHC were offered, e.g., occupational health care for employees working at VDU workstations.

Others said that they were aware of the offer but rejected the opportunity. Other participants considered occupational health care to be beneficial and made use of it. One participant stated that the mode of the invitation might also have an impact:

*“But I would also evaluate, which signs the employer is sending, if there is a note at the notice board saying: You can receive a voluntary OHC during this and this time, or if…the invitation is more insistent or how this is presented”*

(FG-B-03)

However, most participants had not yet been in contact with an OHP at their current workplace and gained new knowledge during the interviews and focus group discussions, e.g., about the existence of OHPs and voluntary OHC.

### 3.5. Research Question 2: Reasons for Not following the Invitation to a Voluntary OHC

#### 3.5.1. Occupational Health Physicians

In response to a standardized question about possible reasons why employees do not accept the invitation to voluntary OHC, nearly 90% of responding OHPs agreed with the statements that employees see no need to consult a physician when they feel healthy and that they prefer their general practitioner or a specialist (see Table 5, items 1 and 2).

Slightly less, but at least 70%, pointed to possible fears among employees that they might suffer internal disadvantages from the results of the OHC in connection with medical confidentiality or data protection.

#### 3.5.2. Employees

As most of the participants had never heard of voluntary OHC before, they discussed expectations rather than experiences. Some preferred to avoid thinking about potential health risks and future health damages that might be associated with their current work. The fear was also expressed that “something” might be found through the voluntary OHC and might corrupt their feeling of healthiness or threaten their current job position.

Some participants did not see the relevance of voluntary OHC for themselves as they felt healthy. Some stated that they saw health-related prevention rather to be connected to private life than occupational life, e.g., in the form of sports or following health nutrition recommendations. Thus, the preventive idea of voluntary OHC either did not fit their concept of prevention or their idea of where prevention should take place. Others expected their employers to take preventive measures to protect their health, but stated at the same time that they would not necessarily make use of it:

*“I realize now that there was actually a voluntary OHC, but I did not go, even though I wish my employer would offer something like this. But I did not go, because I did not have a current problem. And I think, I would go, if I really had some problem, which is actually stupid”*

(FG-B-03, 220)

Some employees did make use of the voluntary OHC because they consulted their own general health physician or specialist and preferred to continue doing so. While some employees reported good experiences with OHPs, most employees voiced concerns about the OHPs’ neutrality and medical confidentiality:

*“For example, in our enterprise, eye examinations are also offered every two years. If I haven’t been to the ophthalmologist for a while, I’ve taken advantage of that in between, because it’s relatively harmless. For really important aspects, I would only go to my family doctor, but not to the OHP, who is paid by the employer. So, I really doubt whether he is always so independent and discreet in individual cases”*

(FG-B-03, 101)

The main reason why employees did not make use of the voluntary OHC was that they did not know anything about the existence of purpose of an OHP.

### 3.6. Research Question 3: Which Aspects Influence the Uptake of Voluntary OHC from a Statistical Point of View Based on the OHP Survey?

In the parsimonious multivariate regression model, the following variables showed a statistical influence (*p* ≤ 0.05) on the assessment by OHPs that employees make use of voluntary OHC always or mostly (sorted by influence power; for more information see Table 6):

- The more positive the attitude of the OHP himself toward voluntary OHC was,
- The more likely it was that employees were invited to voluntary OHC in a targeted manner rather than an untargeted manner,
- The more the invitation to a voluntary OHC was combined with other health care offers (e.g., mandatory OHC, health check-ups),
- Zhe more positively the respondents considered their own role as OHP in the enterprise,
- The fewer details were provided to the employer about the appointment, and
- The longer the OHP worked with the enterprise (as indicator for a relationship of trust between OHP and employees).

The variables “impact of the general economic situation on the enterprise in the last two years” and “importance of occupational health and safety in the enterprise’ subscale ‘enterprise norms”, which showed a significance level between *p* ≥ 0.05 and *p* < 0.2 in the prior bivariate regression analysis, were excluded from the final model. Details of further variables found not to be significant in the prior analysis can be found in Appendix A Table A3. This concerned the following:

1. A spatial proximity between OHP and employees and a generally low-threshold access to the OPH for employees (local accessibility of the OHP´s practice and possibility of spontaneous consultation),
2. The information given in the invitation to voluntary OHC (explicit emphasis of medical confidentiality already in the invitation, number of other important framework conditions mentioned in the invitation letter), and
3. Diverse enterprise characteristics (OHS standards and activities score, explicit general information for voluntary OHC in the enterprise in occupational health and safety instructions/operating instructions, enterprise size).

## 4. Discussion

We reported on the answers of occupational health physicians (OHPs) and employees assessed by a multi-perspective approach researching the uptake of occupational health care as one of the four pillars of workplace health management. The aim of our study was to clarify the extent to which occupational health care, which is voluntary for employees with defined health hazards such as VDU exposition (voluntary OHC), was used and which factors may act as enablers or barriers. This was done by using standardized assessments by occupational health physicians (i.e., an external perspective with regard to the employees) and employees’ self-assessments during the course of qualitative interviews.

Three quarters of the OHPs surveyed in 2012 felt that the majority of employees made use of voluntary OHC when it was offered by the employer. Combining the invitation with other health offers, e.g., *mandatory* occupational health care (which is normal in German OHPs’ daily practice for organizational reasons), seems to encourage the uptake significantly. This was also the third most important predictor for uptake in the multivariate analysis from the OHPs’ point of view.

The question about the uptake did not include a differentiation between occupational activities or exposures based on which voluntary OHC was offered to the employees. In a Dutch survey study, OHPs indicated that employers were not always positive towards workers´ health surveillance [10]. The authors concluded the need to support employers by implementing WHS to increase the provision and uptake of WHS [10]. This corresponds with the results of a survey among German employers on workplace health management showing that OHC was not offered in all enterprises taking part in the survey [1]. The fact that the employers´ attitudes toward occupational safety and health according to corporate social responsibility were revealed to be rather positive in the same survey [31] can be used as starting point to improve the delivery and acceptance of voluntary OHC in the enterprises.

With regard to the general level of uptake, it should be kept in mind that the enterprises were reported by OHPs to have well-organized occupational health and safety management. We obtained less positive results from an analogous survey of employers (*n* = 477, response rate = 21%) [25]. Voluntary OHC was offered in 79% (*n* = 361) of cases, and from these, it was used by the employees in 66% of cases “mostly/always”. Again, almost 90% were representatives of medium and large enterprises with a similar distribution of production/processing industry. One-third was from the public administration sector, while in the OHPs survey, 20% of the enterprises were located in the health care sector. Health care workers know mandatory OHC, which might also make them more familiar with the principles of voluntary OHC. A diverse branch composition in the survey samples might explain the (significant, albeit slight) difference.

In small and medium-sized enterprises, where the occupational health and safety knowledge of employers and the implementation of occupational health and safety measures is significantly lower [32,33], voluntary OHC offers might also be rarer.

Many of the interviewed employees in the qualitative part of the study had never had contact with an OHP, and consequently, most of them were not aware of the relevant terms. This seems to be a reflection of reality. In a recent German survey, 52% of 3039 employees had already had personal contact with an OHP [14]. The interviewed employees indicated that, from their point of view, their acceptance of the employer’s invitation to make use of a voluntary OHC may well depend on the following:

1. Their own assessment of the risk potential of their occupational activity, and
2. The expected consequences of a medical examination.

The first point relates to the fact that it is essential for employees to be well-informed about the risks at their workplace [34], about preventive measures, and about the value of the early detection of possible work-related disorders. This also applies to workplaces where hazardous exposure limits are observed. The second point concerns the fear of a breach of medical confidentiality by the OHP and possible personal disadvantages at the workplace. This fear was also clearly identified by the OHPs in our survey. According to a Dutch study, overall, health checks in the occupational setting are considered interference in one’s personal life, which makes employees rather ambivalent about such health checks [35].

This emphasizes that employees may need to be (even) more clearly informed about the tasks and goals of OHPs in primary and secondary prevention. This information can be practiced, e.g., within the framework of workplace health promotion [36], or as part of the annual occupational safety instructions, or prevention events (action days) or prevention training. These should also involve the executives and, as an incentive, could be certified as further education. Such interventions can help to increase the work-related health literacy and empowerment of employees [23,36]. We know from studies in primary prevention that health literacy has a positive relationship with uptake of screening [37,38].

Finally, a good relationship between employees and OHPs seems to be crucial for the uptake of voluntary OHC [39]. This is also confirmed by the OHP survey results; the (strongest) predictive value in the regression analysis was their own perceived role and the positive effect of a longer duration of the appointment of an OHP at the enterprise. The result is also in line with the OHPs’ contemporary role model, as was retrieved from the items in Appendix A Table A1. The impact of the OHP´s own role and especially of the self-perception of occupational health services on the provision of OHC is supported by findings from a qualitative study among board members of Dutch occupational health services in which several hindering and fostering factors were described [40].

Employees with chronic diseases and those with other health problems or lifestyle risks in particular seem to make use of occupational health services comparatively more often than healthy workers [41,42]. Thus, it is plausible that the most frequently attributed reasons in the external assessment by OHPs were that employees saw no need to consult a physician when they felt healthy and that they preferred to consult their general practitioner or specialist. The latter seems to be different in Finland, for example, where the occupational health physician often replaces visits to the general practitioner’s practice [43]. Improved collaboration between OHPs and general practitioners could also help to clarify the role of OHPs to workers [44,45].

The qualitative part of our study made it clear that employees are often unfamiliar with the terminology used in occupational health and safety, which can have a negative impact on their acceptance of the employer’s offer of occupational health care. This was also confirmed by a study in small German enterprises published in 2014 [46]. Countering the low level of well-informed employees (and employers) first requires sound information strategies in simple language (e.g., [4]). On the other hand, we identified the *targeted* addressing and invitation of employees as the second strongest predictor for the uptake of voluntary OHC from the occupational health physician’s perspective. Even at this point, the invitees should be informed transparently about the aims and framework conditions of occupational health care. This information should include that the employee will not suffer any disadvantages in the case of rejecting the offer, that the costs will be covered by the employer, or that the employer will not receive any information about the result of the appointment [47]. When we look at the OHPs’ answers, there is still room for improvement.

Finally, the content of occupational health care should be designed so that employees experience it as appropriate to their job and helpful in strengthening work-related health. A Dutch intervention study focusing on workers´ health surveillance for workers in the construction industry revealed positive effects of a job-specific WHS in the sense that it aided OHPs in providing workers with recommendations and workers in undertaking preventive action [48]. Similarly, a WHS program designed specifically to address work-related aspects of hospital physicians showed good feasibility and acceptability among participating hospital physicians as well as occupational health professionals and employers (medical managers) [49]. Findings from a recent study from Sweden imply that a meaningful embedding of occupational health care in a comprehensive occupational health and safety system securing the interconnection between the exposure assessment and the medical health check [50] can be perceived as an added value by company representatives [51].

## 5. Conclusions

Voluntary occupational health care seems to be accepted by the majority of employees at least in large German enterprises with good occupational health and safety (OHS) management. More research should be done to gain information in enterprises with lower OHS standards, especially in small and medium-sized enterprises. In this field, employers should be better informed and convinced about possible benefits of occupational health care with regard to the individual employee’s work ability as well as to the general improvement of occupational health and safety.

In the vocational context, the training of other actors such as work councils but also occupational health physicians could be helpful to improve the effective communication with employees about the purpose and benefits of voluntary health screening. General practitioners should be made aware of the work of occupational physicians to encourage employees to use employers’ health offers.

To increase the uptake of existing voluntary OHC, employees should also generally be informed more clearly and in a way that is more appropriate to the target group about the purpose, content, possibilities, and limits of OHC within the context of their occupational activities. In particular, compliance with data protection and medical confidentiality by OHPs should always be emphasized.

As OHC is one important part of workplace health management, providing employees with better information about this service seems essential to increasing their work-related health literacy. Special attention should be paid to target the group-specific design of information about the value of vocational preventive health offers related to specific work hazards.

We assume and hope that the increased health literacy within the population caused by the COVID-19 pandemic since 2020 and the respective involvement of OHPs in broad vaccination offers at the workplace has also brought positive changes in the awareness of employees and employers regarding “traditional” occupational health care.

## 6. Strengths and Limitations of the Study

The strength of the occupational health physicians survey is the sequential mixed-methods approach, which allowed us to develop a questionnaire on the basis of qualitative data and literature reviews, with the consideration of various industrial sectors and regions, and the extent of the data collected, which allowed for multivariate analyses. The limitations of our approach are presented below.

- A questionnaire response rate of only 29% was obtained in the OHP survey. Thus, a sampling bias cannot be ruled out, even if this proportion can in fact be regarded as “within the bounds of what can be expected” for a survey among OH physicians [28,52].
- A data quality limitation is apparent related to the standardized specified reasons for an important outcome, namely the lack of voluntary OHC uptake due to missing values. This confirms the limited informative value of external assessments and points to the need for a survey of employees as mentioned above. It is also questionable to which extent the OHPs who were not employed by the enterprise knew the employees well enough to make a valid assessment of the reasons for lacking voluntary OHC uptake in the enterprise to which the information provided in the survey referred. This was true for two-thirds of our sample, which is in line with the general trend in occupational medicine in Germany.
- A possible positive selection of enterprises in the OHP survey may have been caused by including mainly medium-sized or large companies. These have comparatively higher occupational health and safety standards than small businesses [1,31,53]. This is in contrast to the small enterprises in which the respondents in the qualitative part of the study were predominantly employed in offices. It can be assumed that the quantitative data overestimate the uptake of voluntary OHC, not least because of the results derived from the external assessments by the OHP (and not, for example, by representatives of the human resources departments or of the works councils).
- Most of the OHPs were from enterprises that offered voluntary OHC. We do not know enough about enterprises in which occupational health care is not offered at all or only to a minimum extent. For example, an analysis of the representative German *lidA* study showed that out of 3.039 full-time employees, only about two-thirds knew that there was an occupational health service in the enterprise [14]. Again, a positive bias must be considered. In another survey of employed persons, the proportion who reported contact with an OHP in the last 12 months was even significantly lower (10% [40]).

A strength of the employees’ focus groups and interviews is the large sample size, which allowed for data saturation [54]. In addition, it was satisfactorily possible to generate groups that were heterogeneous within themselves in terms of age, sex, and professional activity. The groups were able to capitalize on the strengths of focus group interviews, which was particularly evident where employees initially appeared to have no knowledge of voluntary OHC but then recalled experiences with them during the course of the discussion. Here, we could benefit from the strength of qualitative methods, which allow comparatively deeper insights compared to quantitative methods [55].

Nevertheless, a standardized survey among employees would have provided a better data basis for statistical analyses and interpretations of employees´ experiences and attitudes toward the uptake of OHC.

Since we could not recruit any production workers, we are also unable to make any respective statements on a qualitative level. Reasons for this lack might be that production workers are more difficult to motivate regarding health and prevention topics, as was also discussed in the focus groups. On the other hand, the homogeneous knowledge deficits of the interviewees are a clearly interpretable result. We know from representative German studies that many employees do not even know their OHP [14], especially if they work in industries in which no mandatory occupational health care must be provided. This also makes our results plausible.

The gap between the OHPs’ assessments and the experiences of the employees we interviewed is obvious. Future studies should close this knowledge gap, e.g., with enterprise-related surveys among employees.

## Figures and Tables

**Table 1:** Characteristics of occupational health physicians (OHPs).

Aspects Described by Percentages		Percent (*n*_(valid)_) ^1^
Occupational health qualification	Specialist in occupational medicine (vs. other (specialist) physician or additional designation “occupational medicine”); *n* = 438	66.7 (438)
Extent of activities as OHP	Full-time (vs. part-time)	78.6 (454)
Number of supervised enterprises	1	15.1 (456)
	2–5	18.2
	6–10	14.9
	11 and more	51.8
Quality management system	In the occupational health physician’s existing practice	43.7 (449)
Extent of activities as OHP	Full-time (35 h/week and more)	60.4 (454)
	Part-time (15–34 h/week)	25.6
	Hourly (14 h/week and less)	13.0
Type of occupational health care ^2^	OHP employed in the enterprise	35.9 (453)
	Own practice (i.e., usually supervising several enterprises)	39.1
	Employed by a commercial inter-company service for OSH	21.3
	Employed by the OHS service of statutory accident insurance	3.7
Gender	Female	45.9 (459)
**Aspects Described by Means**		***M*** **(*SD*)**
Demographic characteristics	Age; *n* = 411	52.7 (6.7)
	Years in current profession; *n* = 446	17.6 (8.0)
	Years of the OHP’s attendance of the enterprise (as indicator for a relationship of trust with employees); *n* = 451	*10.4 (7.7)*
Perceptions	Role of the OHP in the enterprise (self-assessment; score); *n* = 448 ^3^	*2.29 (0.63)*
	OHP’s attitude toward voluntary OHC (score); *n* = 413 ^4^	*1.70 (0.31)*

Notes: *M* = mean; *n* = numbers; OSH = occupational safety and health; *SD* = standard deviation. ^1^ Data base: *n* = 460; ^2^ Indicator for greater spatial proximity to employees; ^3^ 12 items, 4 point Likert-scaled from 0 = “not at all true” to 3 = “totally true”; ^4^ 20 items 4 point Likert-scaled from 0 to 3 (the higher the value, the better the attitude).

**Table 2:** Frame conditions of occupational health and safety in the described enterprise.

Aspect	Percent (*n*_(valid)_)
Type of occupational health care (employment contract between OHP and enterprise vs. supervision by external service; indicator for greater spatial proximity to employees)	35.9 (437)
Type occupational safety specialist supervision (see above)	76.8 (431)
Safety delegates existing (quality of occupational health and safety)	98.0 (450)
Occupational health and safety committee existing	97.4 (454)
Health circle existing	61.2 (443)
Return to work management existing	84.9 (444)
Workplace health promotion offered in the last two years	79.0 (429)
Implementation of mandatory OHC in the enterprise	93.8 (454)
Systematic risk assessments existing	98.7 (450)
Activities for demographic stability addressing employees >50 years of age existing	48.8 (453)
Supportive advice from a supervisor of the statutory accident insurance in the last two years	79.1 (441)
Possibility for employees to consult the OHP’s practice spontaneously (vs. consultation only with prior appointment)	55.3 (406)
Local accessibility of the OHP’s practice −> located in the enterprise/max. 2 km distance vs. farer away)	79.7 (428)
	***M*** **(*SD*)**
Importance of occupational health and safety in the enterprise (subscale “enterprise norms”); *n* = 445 ^1^	2.47 (0.63)
OHS standards and activities (score); *n* = 380 ^2^	7.47 (1.52)

Notes: *M* = mean; *n* = numbers; OSH = occupational safety and health; OHP = occupational health physician. ^1^ 9 items, 5-point Likert-scaled from 0 = ‘totally true/always’ to 4 = not at all ‘true/never’. ^2^ Mean score ‘OHS standards and activities’, possible range from 0 (no respective standards and activities) to 9 (all analyzed standards and activities fulfilled). Items included: Existence of quality management system, works council/staff council, systematic risk assessments, occupational health and safety committee, return to work management, activities for demographic stability addressing employees older than 50 years, safety delegate, health circle and workplace health promotion offered in the last two years.

**Table 3:** Details about how employees are informed about voluntary occupational health care.

		Percent (*n*_(valid)_) ^1^
Communication strategies in general	General reference to occupational health care in occupational safety instructions	80.6 (400)
	General reference to occupational health care in operational instructions	80.6 (400)
Invitation strategies (transparency about important frame conditions of voluntary OHC); average number of items = 3.54 (*SD* 1.9)	Targeted invitation to voluntary OHC (addressed individually vs. untargeted communication in the enterprise) ^2^	94.9 (450)
	Invitation to voluntary OHC in combination with other health care offers ^3^	88.5 (410)
	Information that the employer is obliged to offer the voluntary OHC ^4^	67.8 (379)
	Information about the nature of the hazard on which the offer is based	68.1 (379)
	Information that the employee will not suffer any disadvantages if the offer is rejected	64.6 (379)
	Information that the costs will be covered by the employer	67.3 (379)
	Information that a certificate about the medical results will be issued	61.7 (379)
	Information that the employer will not receive any information about the result of the health care	55.4 (379)
	Explicit emphasis of obligation for professional secrecy already in the invitation (vs. only during appointment or other/no standards)	31.9 (408)
Information provided to the employer	Information provided to the employer only about performed health care, but not about results or these only with agreement of the employee (vs. no information or only statistics)	69.1 (439)

Notes: OHC = occupational health care; OPH = occupational health physician; *SD* = standard deviation; voluntary OHC = voluntary occupational health care. ^1^ Data base: *n* = 460; ^2^ e.g., personal letter/e-mail or oral communication vs. e.g., postings/intranet, circulars, business newspaper, information during occupational health and safety instructions; ^3^ E.g., mandatory OHC, health check-ups; vs. exclusively separate from other; ^4^ Out of *n* = 379 with at least one naming.

**Table 4:** Uptake of voluntary occupational health care by employees, if provided by employer—assessment of occupational health physicians.

Voluntary OHC	Answer Categories	Percent (*n*)
**Uptake**; *n*_(valid)_ = 420 ^1,2^	Always	7.1 (30)
	Mostly	71.2 (299)
	Rarely	21.4 (90)
	Never	0.2 (1)
Uptake if the employee was called simultaneously to mandatory occupational health care; *n*_(valid)_ = 335 ^3^	Markedly higher	66.6 (223)
	Slightly higher	19.4 (65)
	Equal	11.0 (37)
	Slightly less	0.9 (3)
	Considerably less	2.1 (7)

Notes: voluntary OHC = voluntary occupational health care. ^1^ The enterprise to which the information in the questionnaire relates; ^2^ *n* = 479 answers from occupational health physicians, in whose enterprises occupational health care was offered; *n* = 59 missing values/“I don’t know” (12.3%); ^3^ *n* = 398 answers from occupational health physicians, in whose enterprises occupational health care was offered and uptake of voluntary OHC was not rated as “always”; *n* = 63 missing values/“I don’t know” (15.8%).

**Table 5:** Possible reasons for lack of uptake of voluntary occupational health care (assessed by of occupational health physicians; ordered according to frequency of answers).

“The Employees…”	Percent “True Completely/Always” or “True Quite/Often” (*n_(valid)_* Answers)
1 See no need to consult a physician if they feel healthy	89.0 (373)
2 Prefer to consult their general practitioner or specialist	86.9 (313)
3 Are not informed enough about sense and purpose	82.1 (363)
4 Fear that they will be at a disadvantage if results are passed on to the employer	72.8 (334)
5 Are uncertain as to whether the results of the investigation will be passed on to the employer	72.8 (323)
6 Fear that the occupational health physician will check their suitability for their job	70.3 (347)
7 Would (even) have to be invited more clearly	70.1 (334)

Notes: Database: Assessment of “uptake of voluntary OHC” in the enterprise if provided by employer mostly/rarely/never—see Table 4. Exclusion of questionnaires with indication “uptake of voluntary OHC always” and the amount of “I don’t know” and missing answers with regard to possible reasons for lack of uptake of voluntary OHC (missing values no. 1,3, and 6: 5–8%; no. 4,5,7: 10–15%, no: 2: 19%).

**Table 6:** Factors influencing the uptake of voluntary OHC from the perspective of occupational health physicians.

Aspect	*B*	*SE* (*B*)	*df*	*p*	*OR* (*CI* 95%)
OHP’s attitude toward voluntary OHC (score) positive	1.05	0.42	1	0.012	2.85 (1.27–6.44)
Targeted invitation to voluntary OHC (addressed individually vs. untargeted communication in the enterprise)	1.04	0.49	1	0.033	2.82 (1.09–7.32)
Invitation to voluntary OHC in combination with other health care offers (e.g., mandatory OHC health check-ups; vs. exclusively separate from other)	0.98	0.50	1	0.049	2.67 (1.00–7.11)
Role of the OHP in the enterprise (self-assessment; score) positive	0.76	0.35	1	0.028	2.13 (1.08–4.20)
Information provided to the employer only about performed health care without any details (vs. no information or only statistics)	−0.65	0.27	1	0.017	1.91 (0.31–0.89)
Years of the OHP’s attendance to the enterprise	0.05	0.02	1	0.012	1.05 (1.01–1.09)

Notes: Multivariate logistic regression analysis (method: backward; *p_(out)_* = 0.6); imputed data; data basis: *n* = 420 enterprises for which information on the offer of voluntary OHC is available; Abbreviations: *B* = Slope coefficient; *df* = degrees of freedom; *CI* = confidence interval; OHC = occupational health care; OHP = occupational health physician; *OR* = odds ratio; *p* = significance; *SE* = standard error; Model parameter: 78.2% correctly classified values; −2LL = 431.7; Nagelkerke *R*^2^ = 0.39; Excluded from the final regression model: Importance of occupational health and safety in the enterprise (subscale ‘enterprise norms’) and Impact of the general economic situation on the enterprise in the last two years (subjective assessment).

## Data Availability

The data are not publicly available.

## Appendix A

**Table A1:** Single items for score constructions—documentation.

Items for Mean Scores
**Mean score “Importance of occupational health and safety in the enterprise” (subscale “enterprise norms” of the scale FAGS)** [27] **^1^** Health is a top priority at the enterprise The enterprise invests a lot in occupational health and safety In my experience, the importance of occupational health and safety is underestimated in the enterprise Managers are not interested in the health of their employees Nothing is done in the enterprise to ensure that employees remain healthy The measures to maintain and promote employee health in the workplace are good The enterprise does not ask whether the working conditions are detrimental to employee health The regulations on occupational health and safety in the enterprise are good What the enterprise does for health is either insufficient or nonsensical
**Mean score “Role of the occupational health physician (OHP) in the enterprise” (self-development) ^2^** The OHP has direct access to the enterprise management The OHP is well-aware of the exact workplace conditions in the enterprise Employees know how to contact the OHP There is good cooperation between the OHP and the occupational safety specialist Cooperation between the OHP and the works council is good The employer likes the measures proposed by the OHP to improve occupational health and safety The OHP’s work is good The OHP is easily accessible for employees The range of tasks for the OHP is clearly defined The OHP has a great scope of action. Employees have a confidential relationship with the OHP The specified deployment times are sufficient to be able to perform prevention tasks appropriately
**Mean score “OHPs’ attitude toward voluntary occupational health care” (self-development**) **^3^** *Consent to positive assessment:* Voluntary OHCs strengthen the self-responsibility of the employee Voluntary OHCs are a low-threshold opportunity to get in touch with the employee Voluntary OHCs are an important source of information for a better assessment of the employee’s workplace Voluntary OHCs help to educate employees about work-related hazards Voluntary OHCs help to allay employees’ fears about hazards in the workplace Voluntary OHCs are an important instrument for raising employees’ awareness of health issues Voluntary OHCs should also be available for employees with high physical stress (lifting, carrying, etc.) Voluntary OHCs should also be available for employees with high psychological stress *Consent to negative assessment:* Voluntary OHCs are not important, other than mandatory OHC (*) Voluntary OHCs cause more concern among employees than the benefit justifies (*) Voluntary OHCs are less useful than offering simple ‘health check ups’ to all employees (*) Voluntary OHCs keep OHPs from other important tasks, e.g., workplace assessments (*) Voluntary OHCs are not necessary for some hazards where they are intended to be used (*) Voluntary OHCs do not precisely address the aspects that are of primary concern to the employees in the company (e.g., mental stress, musculoskeletal complaints, etc.) (*) Medical confidentiality complicates the proof of the action of occupational health physicians towards the employer (*) *Restrictive assessment/situation description:* Voluntary OHCs are only possible if the enterprise has an economically good standing(*) Voluntary OHCs depend on the personal engagement of the OHP (*) Voluntary OHCs would not even be possible in many enterprises if all employees were to take part in them (*) Voluntary OHCs are only useful if hazards at the workplace are also identified and eliminated/minimized (*) Voluntary OHCs are not useful if the employer cannot draw any conclusions from the results for the design of workplaces (*)

Notes: Voluntary OHC = voluntary occupational health care; (*) Negative Item wording; inverted for score construction; ^1^ Nine items, 5-point Likert scale from 0 = “totally true /always” to 4 = “not at all true/never”; ^2^ 12 items, 4 point Likert scale from 0 = “not at all true” to 3 = “totally true”; items no. 9–20 inverted for score construction; ^3^ 20 items, 4 point Likert scale from 0 = “not at all/never true” to 3 = “absolutely/always true”.

**Table A2:** Key questions for employees in interviews and focus group discussions.

**Introduction: How is the issue of health dealt with in the company you work for?**
- How important is the topic of health in your enterprise? - What do you do for your health at work? - Who do you turn to at work when you have health questions?
**Transition: What experiences have you had with OHPs so far?**
- Why did you go to the OHP? - Have you ever refused medical examinations? - Does a visit to the OHP make sense at all? - What do you think how you receive the results?
**Main part**
- Have you ever heard of the term “voluntary occupational health care”? - Your enterprise is required by law to offer you preventive medical checkups as part of occupational health and safety, for example, for certain hazardous substances, work at computer screens, or work with moisture. These examinations are mandatory for you if certain limit values are exceeded. This is called “mandatory occupational health care”. The health care is voluntary for you if certain limit values are not reached. This is called “voluntary occupational health care”. What do you think of that? - Under what circumstances would you or other employees see the OHP for voluntary occupational health care? - What can the enterprise do to persuade you to make use of voluntary occupational health care? - What can the OHP do to persuade you to make use of voluntary occupational health care? - What keeps you or other employees from going to occupational health examinations? - You have now mentioned a few difficulties. Who could do something about it? - What concerns do you have about participating in health screenings by the company physician?
**Conclusion**
- Looking at our conversation and your experience now, what would it take for you or other employees to take advantage of voluntary occupational health examinations by the OHP company physician?

Notes: OHP = occupational health physicians.

**Table A3:** Possible predictors tested exploratively on outcome “uptake of voluntary occupational health care” in bivariate logistic regression analysis.

Characteristics of…	Aspect	Data Level	*p* ^1^
1. Frame conditions in the enterprise for occupational health and safety (OHS)	Importance of occupational health and safety in the enterprise (subscale “enterprise norms”) ^2^	M	*
	OHS standards and activities (score) ^3^	M	---
	Type of occupational health care (employment contract between OHP and enterprise vs. supervision by external service) ^4^	D	---
2. Occupational health care (OHC) characteristics	Years of the OHPs’ attendance of the enterprise ^5^	M	***
	Possibility for employees to consult the OHPs’ practice spontaneously (vs. consultation only with prior appointment) ^6^	D	---
	Local accessibility of the OHPs’ practice (in the enterprise/max. 2 km distance) ^6^	D	---
3. Characteristics of information management about voluntary OHC	Targeted invitation to voluntary OHC (addressed individually vs. untargeted communication in the enterprise)	D	***
	Invitation to voluntary OHC in combination with other health care services (e.g., mandatory OHC, health check-ups; vs. exclusively/separate from other)	D	***
	General reference to occupational health care in occupational safety instructions vs. no/not known)	D	---
	General reference to occupational health care in operating instructions (vs. no/not known)	D	---
	Transparency about the nature of voluntary OHC (number of respective important frame conditions mentioned already in the invitation) ^7^	M	---
	Explicit emphasis of obligation for medical confidentiality already in the invitation (vs. only during appointment or other/no standards)	D	---
	Information provided to the employer about performed health care without any details or details only with the employees’ agreement (vs. no information or only statistics)	D	***
4. Perceptions of occupational health physicians (OHPs)	Role of the OHP in the enterprise (self-assessment; score) ^8^	M	***
	OHP’s attitude toward voluntary OHC (score) ^9^	M	***
5. Enterprise characteristics	Impact of the general economic situation on the enterprise in the last two years (subjective assessment) ^10^	C	*
	Large-scale enterprise (251 or more employees vs. medium-sized company, 51–250 employees)		---

Notes: C = categorical variable (−1 negative, 0 no change, 1 positive (reference category)); D = dichotomous variable (1 = applies, 0 = applies not); M = metric variable; OHC = occupational health care; OHP = occupational health physician; *p* = significance; *SD* = standard deviation; voluntary OHC = voluntary occupational health care; ^1^ Imputed data, *n* = 460, *n* = 5 imputations, automatic method; bivariate linear regression analysis prior to multivariate model; *** = *p* ≤ 5% (significant); * = *p* ≥ 5% and <20% (conspicuous result); ‘---’= *p* ≥ 20% (not significant at all); variables sorted by significance; ^2^ 9 items, 5-point Likert-scaled from 0= ‘totally/always true’ to 4 =‘not at all/never’ true; ^3^ Mean score ‘OHS standards and activities’, possible range from 0 (no respective standards and activities) to 9 (all analyzed standards and activities fulfilled). Items included depicting OHS standards and activities: Existence of quality management system, works council/staff council, systematic risk assessments, occupational health and safety committee, return to work management, activities for demographic stability addressing employees older than 50 years, safety delegates, health circle, and workplace health promotion offered in the last two years; ^4^ Indicator for stronger presence in the enterprise compared to a supervision of the enterprise by an external OHC service. ^5^ Indicator for a possible relationship of trust between occupational health physician and employees; ^6^ Indicator for low-threshold offer; ^7^ Six items: 1. Information that the employer is obliged to offer the voluntary OHC (67.8% out of *n* = 379 with at least one naming), 2. Information about the nature of the hazard on which the offer is based (68.1%), 3. Information that the employee will not suffer any disadvantages if the offer is rejected (64.6%), 4. Information that the costs will be covered by the employer (67.3%), 5. Information that a certificate about the medical results will be issued (61.7%), and 6. Information that the employer will not receive any information about the result of the appointment (55.4%); possible range 0–6; ^8^ 12 items, 4 point Likert scale from 0 = “not at all true” to 3 = “totally true”; ^9^ 20 items, 4 point Likert scale from 0 to 3, partly inverted (see Table A1 above); the higher the value, the more positive the attitude; ^10^ 7 point Likert scale from “very, very negative” (−1 to −3), 0 “no change” to “very, very positive” (+1 to +3) categorized into −1 (negative impact), 0 (no change) and 1 (positive impact).

**Table A4:** Characteristics of the described enterprise.

Aspect	Percent (*n*_(valid)_)
Large-scale enterprise (251 employees and more; vs. medium-sized enterprises, 51–250 employees) ^1^	95.4 (439)
**Economic sector (*n* = 421)**	
Industry (production/processing—metal and electrical)	39.4
Industry (production/processing—glass, ceramics, wood, paper, food, printing)	7.8
Health service	20.4
Welfare (disabled facilities, child daycare, schools)	2.9
Construction/mining	5.2
Agriculture/forestry	0.2
Public administration	8.1
Utilities and waste management	4.5
Transport/logistics	3.1
Service (focus on office)	7.8
Service (focus on cleaning)	0.5
Works council/staff council existing	95.2 (455)
Quality management system existing	87.5 (455)
Negative impact of the general economic situation on the enterprise in the last two years ^2^	47.6 (441)

^1^ Categorized by Commission Recommendation of 6 May 2003 concerning the definition of micro, small and medium-sized enterprises (Text with EEA relevance) (notified under document number C(2003) 1422) https://eur-lex.europa.eu/eli/reco/2003/361/oj (accessed on 20 June 2022); ^2^ Subjective assessment; 7 point Likert scale from “something to very, very negative” (−1 to −3) to “something to very, very positive” (+1 to +3); categorized; mean value (uncategorized) *M* = −0.22 (*SD* 0.07).

**Table A5:** Characteristics of Employees in focus group discussions and expert interviews.

Transcript No.	Sex	Age Group (Year)	Profession/Professional Activity	Working in…
FG B-01	f	40–50	Clerk	Private sector
	f	20–30	Administration	Public sector
	m	50–60	Letter carrier	Public sector
	f	20–30	Retail clerk	Private sector
	f	30–40	Lawyer	Private sector
FG B-02	f	30–40	Medical technical assistant	Public sector
	f	40–50	Therapist	Public sector
	f	40–50	Medical technical assistant	Public sector
	m	40–50	Computer scientist	Private sector
FG B-03	m	20–30	Technical assistant	Private sector
	m	50–60	Surveyor technician	Private sector
	f	50–60	Quality control nurse	Private sector
	f	20–30	Flight attendant	Private sector
	f	30–40	Clerk	Public sector
	m	50–60	Bank clerk	Private sector
FG B-04	m	40–50	Engineer	Industry
	m	40–50	Engineer	Industry
	f	50–60	Administrative job	Public sector
	m	40–50	Photographer	Self-employed
FG B-05	f	40–50	Home and youth educator	Private sector
	m	30–40	Archaeologist	Public sector
	f	50–60	Clerk	Craft business
EI-14	m	40–50	Bank clerk	Private sector
EI-16	m	30–40	Biologist	Public sector

Notes: EI = expert interview; f = female; FG = focus group; m = male.

**Table A6:** Exemplary quotations from employees in interviews and focus discussions to research question 1: Uptake of voluntary occupational health care.

Summary	Citations
Most employees had not heard of the terms before (interviewer (I), employee (E))	(I): Have you ever heard of the term voluntary occupational health care? (E1): No. (E2): Voluntary occupational health care? Now—also in the context of occupation or at the primary care physicians or specialists? (I): By occupational health physician. (E3): From the occupational health physician. Voluntary occupational health care? (E4): We have this—not with that term—but I imagine that it is just an offer like, for example, now the vision exam... vision check, yes, or flu vaccination. You can accept it for whatever reason it’s offered, but you don’t have to, right?
Nevertheless, some participants remembered voluntary OHC during the course of the discussion and reported that they actually have had voluntary OHC without knowing what it is concretely	For example, in our enterprise, eye examinations are offered every two years. If I haven’t been to the ophthalmologist for a while, I’ve taken advantage of that in between, because it’s relatively harmless. For really important aspects, I would only go to my general physician, but not to the occupational health physician, who is paid by my employer. So, whether he is always so independent and discreet in individual cases, that’s just too unclear to me (FG-B-03, 101)

Notes: E = interview; FG = focus group discussion.

## References

[1] Hoge A., Ehmann A.T., Rieger M.A., Siegel A. (2019). Caring for Workers’ Health: Do German Employers Follow a Comprehensive Approach Similar to the Total Worker Health Concept? Results of a Survey in an Economically Powerful Region in Germany. Int. J. Environ. Res. Public Health.

[2] Los F.S., de Boer A.G.E.M., van der Molen H.F., Hulshof C.T.J. (2019). The Implementation of Workers’ Health Surveillance by Occupational Physicians: A Survey Study. J. Occup. Environ. Med..

[3] Rantanen J. (2005). Basic occupational health service—Their structure, content and objectives. SJWEH Suppl..

[4] Guidotti T.L. Creating a Safe and Healthy Workplace. A Guide to Occupational Health and Safety for Entrepreneurs, Owners and Managers. Version 6. Commission on Occupational Health (ICOH) 2014. http://www.icohweb.org/site/oh-guide.asp#download.

[5] Colosio C., Mandic-Rajcevic S., Godderis L., van der Laan G., Hulshof C., van Dijk F. (2017). Workers’ health surveillance: Implementation of the Directive 89/391/EEC in Europe. Occup. Med..

[6] International Labour Organization (1998). Technical and Ethical Guidance Documents for Worker’s Health Surveillance. Occupational Safety and Health Eries 72.

[7] Koh D., Aw T.C. (2003). Surveillance in occupational health. Occup. Environ. Med..

[8] Federal Ministry of Labor and Social Affairs Occupational Health Care Ordinance of 18 December 2008, Federal Law Gazette I, p. 2768, as Last Amended by Article 1 of the Ordinance of 12 July 2019 (Federal Law Gazette I, p. 1082). https://www.gesetze-im-internet.de/englisch_arbmedvv/englisch_arbmedvv.html.

[9] Berger J., Grabbe Y., Loos S., Nolting H.-D., Will N., Matschke B. (2005). Occupational Health Screening in Six Countries of the European Union. Evaluation with Regard to Innovative Concepts of Individual Occupational Medical Prevention [German: Arbeitsmedizinische Vorsorgeuntersuchungen (AMVU) in Sechs Ländern der Europäischen Union. Bewertung im Hinblick auf Innovative Konzepte der Arbeitsmedizinischen Individualprävention].

[10] Los F.S., van der Molen H.F., Hulshof C.T.J., de Boer A.G.E.M. (2021). Supporting Occupational Physicians in the Implementation of Workers’ Health Surveillance: Development of an Intervention Using the Behavior Change Wheel Framework. Int. J. Environ. Res. Public Health.

[11] Barth C.H., Hamacher W., Eickholt C., Federal Institute for Occupational Safety and Health (BAuA) (2014). Demand for Occupational Health Care in Germany.

[12] Hulsegge G., Proper K.I., Loef B., Paagman H., Anema J.R., van Mechelen W. (2021). The mediating role of lifestyle in the relationship between shift work, obesity and diabetes. Int. Arch. Occup. Environ. Health.

[13] Krogsbøll L.T., Jørgensen K.J., Gøtzsche P.C. (2019). General health checks in adults for reducing morbidity and mortality from disease: Cochrane systematic review and meta-analysis. Cochrane Database Syst. Rev..

[14] Hasselhorn H.-M., Michaelis M., Kujath P. (2020). Occupational health provision among workers in Germany: Results of the representative lidA study. Arb. Soz. Umw..

[15] Robert Koch-Institute (2017). Utilization of healthcare and preventive healthcare in Germany of Health Monitoring. JoHM.

[16] Lederle M., Tempes J., Bitzer E.M. (2021). Application of Andersen’s behavioural model of health services use: A scoping review with a focus on qualitative health services research. BMJ Open.

[17] SoleimanvandiAzar N., Mohaqeqi Kamal S.H., Sajjadi H., Ghaedamini Harouni G., Karimi S.E., Djalalinia S., Setareh Forouzan A. (2020). Determinants of Outpatient Health Service Utilization according to Andersen’s Behavioral Model: A Systematic Scoping Review. Iran. J. Med. Sci..

[18] Kurspahić-Mujčić A., Mujčić A. (2019). Preventive health services utilization in patients treated by family physicians. Med. Glas..

[19] De Boer A.G.E.M., Wijker W., de Haes H.C.J.M. (1997). Predictors of health care utilization in the chronically ill: A review of the literature. Health Policy.

[20] Plomp H.N. (1998). The intention to utilize occupational health services. Occup. Med..

[21] Harkko J., Sumanen H., Pietiläinen O., Piha K., Mänty M., Lallukka T., Rahkonen O., Kouvonen A. (2020). Socioeconomic Differences in Occupational Health Service Utilization and Sickness Absence Due to Mental Disorders: A Register-Based Retrospective Cohort Study. Int. J. Environ. Res. Public Health.

[22] Schubin K., Schlomann L., Lindert L., Pfaff H., Choi K.E. (2020). Occupational Physicians’ Perspectives on Determinants of Employee Participation in a Randomized Controlled Musculoskeletal Health Promotion Measure: A Qualitative Study. Int. J. Environ. Res. Public Health.

[23] Sigblad F., Savela M., Okenwa Emegwa L. (2020). Managers’ Perceptions of Factors Affecting Employees’ Uptake of Workplace Health Promotion (WHP) Offers. Front. Public Health.

[24] Reinhardt A., Adams J., Schöne K., Rose D.M., Sammito S. (2020). Do working characteristics influence the participation at health measures? Findings from a trial phase of workplace health promotion. J. Occup. Med. Toxicol..

[25] Völter-Mahlknecht S., Michaelis M., Preiser C., Blomberg N., Rieger M.A., Federal Ministry of Labor and Social Affairs (BMAS) (2014). Uptake of Voluntary Occupational Health Care Offered in the Occupational Health Care System.

[26] Moore G.F., Audrey S., Barker M., Bond L., Bonell C., Hardeman W., Moore L., O’Cathain A., Tinati T., Wight D. (2015). Process evaluation of complex interventions: Medical Research Council guidance. BMJ.

[27] Stapp M. (2018). Occupational Health and Safety Questionnaire (FAGS). An Instrument for Assessing Occupational Health and Safety Management in Industrial Companies.

[28] Nübling M., Lincke H., Wahl-Wachendorf A., Jurkschat R., Panter W. (2014). Psychosocial working conditions, stress and health behaviour of occupational health physicians. Arbeitsmed. Sozialmed. Umw..

[29] German Professional Association of German Occupational Health Physicians. https://www.vdbw.de/.

[30] Schreier M. (2013). Qualitative analysis methods. Research Methods in Psychology and the Social Sciences for Bachelor.

[31] Siegel A., Hoge A.C., Ehmann A.T., Martus P., Rieger M.A. (2021). Attitudes of Company Executives toward a Comprehensive Workplace Health Management—Results of an Exploratory Cross-Sectional Study in Germany. Int. J. Environ. Res. Public Health.

[32] Amler N., Voss A., Wischlitzki E., Quittkat C., Sedlaczek S., Nesseler T., Letzel S., Drexler H. Implementation of Relevant Regulations Concerning Occupational Safety and Health—Status Quo, Level of Knowledge and Information and Assistance Needs in Small and Medium-Sized Enterprises (SME). https://www.gesund-arbeiten-in-thueringen.de/fileadmin/pdf/Publikationen_GAIT/ASU_2019-01_GAIT_Unternehmensbefragung_Amler_Voss_etc.pdf.

[33] European Agency for Safety and Health at Work (2018). Safety and Health in Micro and Small Enterprises in the EU: Final Report from the 3-Year SESAME Project.

[34] Hamacher W., Eickholt C., Lenartz N., Blanco S., Federal Institute for Occupational Safety and Health (BAuA) (2012). Safety and Health Competence through Informal Learning in the Work Process.

[35] Damman O.C., van der Beek A.J., Timmermans D.R. (2015). Employees are ambivalent about health checks in the occupational setting. Occup. Med..

[36] Ehmann A.T., Ög E., Rieger M.A., Siegel A. (2021). Work-Related Health Literacy: A Scoping Review to Clarify the Concept. Int. J. Environ. Res. Public Health.

[37] Oldach B.R., Katz M.L. (2014). Health literacy and cancer screening: A systematic review. Patient Educ. Couns..

[38] Lee H.Y., Kim S., Neese J., Lee M.H. (2021). Does health literacy affect the uptake of annual physical check-ups?: Results from the 2017 US health information national trends survey. Arch. Public Health.

[39] Amler N., Nesseler T., Letzel S., Drexler H. Occupational Health Care by Means of Telemedical Procedures—Results of a Study from the Employees’ Point of View. Proceedings of the German Society for Occupational and Environmental Medicine (DGAUM): The 61st Annual Meeting 2021.

[40] Los F.S., Hulshof C.T.J., Sluiter J.K. (2019). The view and policy of management of occupational health services on the performance of workers’ health surveillance: A qualitative exploration. BMC Health Serv. Res..

[41] Schnee M., Mosebach K., Groneberg D.A. (2012). What kind of employees contact the company doctor?. Zent. Arb. Arb. Ergon..

[42] Reho T., Atkins S., Korhonen M., Siukola A., Sumanen M., Viljamaa M., Uitti J., Sauni R. (2021). Sociodemographic characteristics and disability pensions of frequent attenders in occupational health primary care—A follow-up study in Finland. BMC Public Health.

[43] Ikonen A., Räsänen K., Manninen P., Rautio M., Husman P., Ojajärvi A., Alha P., Husman K. (2013). Use of health services by Finnish employees in regard to health-related factors: The population-based Health 2000 study. Int. Arch. Occup. Environ. Health.

[44] Moßhammer D., Michaelis M., Mehne J., Wilm S., Rieger M.A. (2016). General practitioners’ and occupational health physicians’ views on their cooperation: A cross-sectional postal survey. Int. Arch. Occup. Environ. Health.

[45] Stratil J.M., Rieger M.A., Völter-Mahlknecht S. (2017). Cooperation between general practitioners, occupational health physicians, and rehabilitation physicians in Germany: What are problems and barriers to cooperation? A qualitative study. Int. Arch. Occup. Environ. Health.

[46] Sczesny C., Keindorf S., Dross P., Jasper G., Federal Institute for Occupational Safety and Health (BAuA) (2014). Level of Knowledge of Enterprises and Employees in the Field of Occupational Health and Safety in Small and Bittel Enterprises.

[47] BMAS—Federal Ministry of Labor and Social Affairs (2014). Occupational Medical Rule 5.1 ‘Requirements for the Provision of Occupational Health Care’.

[48] Boschman J.S., Van der Molen H.F., Frings-Dresen M.H., Sluiter J.K. (2013). Preventive actions taken by workers after workers’ health surveillance: A controlled trial. J. Occup. Environ. Med..

[49] Ruitenburg M.M., Plat M.C., Frings-Dresen M.H., Sluiter J.K. (2015). Feasibility and acceptability of a workers’ health surveillance program for hospital physicians. Int. J. Occup. Med. Environ. Health.

[50] Eliasson K., Palm P., Nordander C., Dahlgren G., Lewis C., Hellman T., Svartengren M., Nyman T. (2020). Study protocol for a qualitative research project exploring an occupational health surveillance model for workers exposed to hand-intensive work. Int. J. Environ. Res. Public Health.

[51] Eliasson K., Dahlgren G., Hellman T., Lewis C., Palm P., Svartengren M., Nyman T. (2021). Company Representatives’ Experiences of Occupational Health Surveillance for Workers Exposed to Hand-Intensive Work: A Qualitative Study. Int. J. Environ. Res. Public Health.

[52] Rothermund E., Michaelis M., Jarczok M.N., Balint E.M., Lange R., Zipfel S., Gündel H., Rieger M.A., Junne F. (2018). Prevention of Common Mental Disorders in Employees. Perspectives on Collaboration from Three Health Care Professions. Int. J. Environ. Res. Public. Health.

[53] Hägele H. (2019). Final Report on the Umbrella Evaluation of the Joint German Occupational Safety and Health Strategy 2nd Strategy Period.

[54] Mey G., Mruck K. (2011). Grounded Theory Reader.

[55] Creswell J.W., Poth C.N. (2016). Qualitative Inquiry and Research Design.

